# Complex interaction networks of cytokines after transarterial chemotherapy in patients with hepatocellular carcinoma

**DOI:** 10.1371/journal.pone.0224318

**Published:** 2019-11-21

**Authors:** Dong Wook Jekarl, Seungok Lee, Jung Hyun Kwon, Soon Woo Nam, Myungshin Kim, Yonggoo Kim, Jeong Won Jang

**Affiliations:** 1 Department of Laboratory Medicine, Seoul St. Mary’s Hospital, College of Medicine, The Catholic University of Korea, Seoul, Korea; 2 Laboratory for Development and Evaluation Center, Seoul St. Mary’s Hospital, College of Medicine, The Catholic University of Korea, Seoul, Korea; 3 Department of Laboratory Medicine, Incheon St. Mary’s Hospital, College of Medicine, The Catholic University of Korea, Seoul, Korea; 4 Department of Internal Medicine, Incheon St. Mary’s Hospital, College of Medicine, The Catholic University of Korea, Seoul, Korea; 5 Department of Internal Medicine, Seoul St. Mary’s Hospital, College of Medicine, The Catholic University of Korea, Seoul, Korea; American Society for Investigative Pathology, UNITED STATES

## Abstract

Treating hepatocellular carcinoma with transarterial chemoembolization (TACE) induces both local inflammation in the tumor microenvironment as well as systemic inflammation. We analyzed serum cytokine response to TACE to evaluate this. Serum samples obtained from 203 HCC patients treated with TACE were analyzed for inflammatory cytokines including interleukin (IL)-1β, IL-2, IL-4, IL-5, IL-6, IL-9, IL-10, IL-12, IL-13, IL-17, IL-22, TNF-α, IFN-γ, and C-reactive protein (CRP) levels. Cytokine concentrations were measured at day 0 (D0, baseline, n = 203), day3 (D3, n = 156), day7 (D7, n = 147), and day 60 (D60, n = 115) after TACE. Network analysis of the cytokines was performed to understand their interactive relationship. After TACE, IL-1β, -6,-9, -12, and -22 increased by D60. IL-2, -5, -10, -17A and INF-γ decreased by D60, and IL-4, -13 and TNF-α revealed stable concentration. D0 network revealed that IL-2, -4, -5, and -10 formed a module. D3 network had the highest clustering coefficient and average degree that revealed similar pattern as CRP. D7 network revealed that IL-6, -9 and CRP were isolated from the network. D60 network had the lower network heterogeneity and lower clustering coefficient, network diameter, shortest path and characteristic path length. Degree correlation revealed that assortative network turned to disassortative network by D60 indicating that the network gained scale free feature. D60 cytokine network retained inflammatory function and these parameters indicated that the systemic inflammation induced by TACE appeared to be attenuated by D60. IL-9 at D3 and D7 seemed to be related to anti-tumor effect and IL-6 at D7 and D60, and IL-22 at D60 was related to regenerative but not pro- or anti- inflammatory function. Median survival month of patient group with high and low values of cytokine with P-values were as follows: D0 CRP, 9.5 and 54.2 months (P<0.0001); D0 IL-2, 39.9 and 56.1 months (P = 0.0084); D3 CRP, 31.3 and 55.1 months (P = 0.0056); D7 CRP, 28.7 and 50.7 months (P = 0.0065), respectively. TACE is associated with systemic inflammation which appears to peak at Day 3 and resolve by D60. Among the tested cytokines, IL-6 and IL-22 appear to play a regenerative role.

## Introduction

Hepatocellular carcinoma (HCC) is the most common malignancy of primary liver cancer, the 6^th^ most commonly diagnosed cancer, and in 2018 was the fourth leading cause of cancer-related death worldwide [1]. In males, HCC is the 5^th^ most common cancer and the 2^nd^ leading cause of death, with an incidence rate twice that of females. Major risk factors for HCC are chronic hepatitis via hepatitis B and C viruses, alcoholic liver disease, aflatoxin B1-contaminated foods, nonalcoholic hepatitis, obesity, smoking, and type 2 diabetes [1, 2].

Pathophysiology of HCC is related to molecular gene alternations and driver mutations in the cell and the microenvironment surrounding the cell [3]; the inflammatory microenvironment caused by viral and toxic risk factors plays an important role in HCC development [4]. These local inflammatory responses have systemic effects through direct release of inflammatory cytokines or indirect alteration in inflammatory homeostasis. These systemic effects results in a resetting of the tissue inflammatory state [5–8]. The Systemic inflammatory response is related to the prognosis of HCC, which includes neutrophil to lymphocyte ratio, platelet to lymphocyte ratio and Glasgow Prognostic Score [7–8].

Inflammation is a complex network that plays an important role in development, progress and migration in HCC [6–7]. Because no single molecule acts as a lone factor for the liver carcinogenesis, complex interaction networks should be considered. A network is comprised of a set of nodes that represent entities such as cytokines, genes, or proteins and a set of edges or links that define the relationships between nodes [9]. The relationship between nodes could be a physical interaction, physical link, or represent mass/energy exchange [9, 10]. Biological network can be composed of clustered molecules with both physical and non-physical interactions. Networks with physical interactions include protein-to-protein interaction networks, and networks with non-physical interactions include co-expression networks, disease networks, molecular pathway interactions, and gene versus phenotypes [11, 12]. A conceptual network of cytokine co-expression data might reveal complex interactions in HCC patients treated with TACE. Therefore, in this study, a systemic inflammation of complex interaction network was explored by comparative analysis of multiple cytokines IL-1β, IL-2, IL-4, IL-5, IL-6, IL-9, IL-10, IL-12, IL-13, IL-17, IL-22, TNF-α, IFN-γ, and C-reactive protein (CRP), measured at baseline (D0), day 3 (D3), day 7 (D7), and day 60 (D60) after TACE.

## Materials and methods

### Patients

This study was approved by the Institutional Review Board of Incheon St. Mary’s Hospital. After informed consent provided from participants, serum samples obtained from 203 HCC patients treated with TACE were analyzed for inflammatory cytokines including interleukin (IL)-1β, IL-2, IL-4, IL-5, IL-6, IL-9, IL-10, IL-12p70, IL-13, IL-17, IL-22, TNF-α, IFN-γ, and CRP levels between June 2011 and December 2012 [13, 14]. For prognostic analysis and estimation of overall survival, patient data were collected by December 2018. Patients with an unresectable tumor graded as Child-Pugh class A or B without evidence of portal vein involvement who were initially treated with TACE were selected [13]. HCC diagnosis was based on histology or elevated α-fetoprotein level in radiologic findings. TACE agents were composed of doxorubicin (50 mg) or epirubicin (50 mg) and cisplatin (60 mg) with lipiodol (5–10 mL) based on baseline tumor extent [13, 14]. Baseline characteristics of the datasets are listed in Table 1.

**Table 1:** Baseline patient characteristics^a^.

	Patient (n = 203)
Age (year)	58 (40–84)
Sex, Male / Female	164 / 39
Etiology, HBV / HCV / Alcohol / Others	142 / 17 / 22 / 22
Ascite, None / Present	172 / 31
Portal hypertension, None / Present	155 / 48
ECOG, 0 / 1 / 2 / 3 ^b^	115 / 49 / 27 / 4
Child Pugh Class, A / B / C ^b^	141 / 49 / 5
Tumor Maximum Size (cm)	5.7 (0.8–28)
Tumor number, single / multiple	102 / 101
Metastasis, None / present	169 / 34
Treatment	
TAC / +PEI / +Radiation / Sorafenib / Others	127 / 3 / 16 / 5 / 52
Laboratory data, median (range)	
Platelet (x10^9^/L)	131 (0.95–502)
Prothrombin time (INR)	1.22 (0.98–2.46)
Bilirubin (mg/dL)	1.2 (0.3–9.7)
AST (U/L)	51.5 (11–522)
ALT (U/L)	36 (11–392)
Albumin (d/dL)	3.6 (0.8–5.1)
Creatinine (mg/dL)	0.7 (0.4–1.3)
Sodium (mmol/L)	138 (125–144)
AFP (ng/mL)	57.6 (1.3–3,000)
PIVKA-II (mAU)	193 (10–75000)

HBV, hepatitis B virus; HCV, hepatitis C virus; TAC, transarterial chemotherapy; PEI, percutaneous ethanol infection; AST, aspartate aminotransferase; ALT, alanine aminotransferase; AFP, alpha fetoprotein; PIVKA-II, protein induced by vitamin K absence or antagonist II.

^a^The continuous variables are presentedas median (range).

^b^For ECOG (Eastern Cooperative Oncology Group) and Child Pugh classification, 8 patients had no data.

### Cytokine measurement

The cytokines tested during TACE were IL-1β, IL-2, IL-4, IL-5, IL-6, IL-9, IL-10, IL-12, IL-13, IL-17, IL-22, TNF-α, and IFN-γ. Cytokines were measured using a Flowcytomix Multiplex with a multiple cytometric bead immunoassay (eBioscience, San Diego, CA, USA) at baseline (D0, n = 203), day 3 (D3, n = 156), day 7 (D7, n = 147), and day 60 (D60, n = 115) after TACE.

### Laboratory data measurements

CRP level, liver panel, and other blood chemistry data were measured at the same time as the cytokines using a Beckman Coulter AU5800 Clinical Chemistry System (Beckman Coulter, Miami, FL, USA). Data related to viral hepatitis were measured using an Architecti2000 analyzer (Abbott Laboratories, Chicago, IL, USA). Platelet counts were measured using an XE-2100 differential analyzer (Sysmex Corporation, Kobe, Japan).

### Network topology analysis

Statistical analyses of cytokine profiles were performed with Spearman’s correlations, and statistically significant cytokine pairs were used for network analysis. Each cytokine was regarded as a node, and correlated pairs were regarded as links (edges) in the network [15]. Cytoscape and NetworkAnalyzer were used for network analyses [16–19]. Degree correlations, modularity and clustering analysis were calculated using iGraph, which is a package of R program [20, 21].

As defined by graph theory, the clustering coefficient, <*C*>, is the number of edges from the nodes (cytokine), connected each other, which all allow for a connection to node *i*. The degree measurement is the number of edges (links or correlation pairs) connected to a node. Network density is the ratio of edges in the network to the total possible number of edges. The length of a path is the number of unique edges that form between two nodes. Distance is defined as the shortest path length between nodes *i* and *j* within the network. Network diameter is the maximum length of the shortest path. The characteristic path length is the average shortest path length that generates expected distance between two nodes, *i* and *j*. The average degree, <*k*>, indicates the average number of edges from the nodes. Network heterogeneity is a measure of variance in the number of edges divided by the mean number of edges. The topological coefficient is a relative measure for the extent to which a node shares a neighbor with another node. The network centralization value approaches 1 when the network resembles a star. Stress centrality of a node is the number of shortest paths passing through the node. Betweenness centrality of node *k* defines the shortest path between nodes *i* and *j* that pass through node *k* and implies that node *k* exerts control over other nodes. Closeness centrality of a node reflects how close it is to other nodes in the network. Eccentricity reflects maximum distance from a node [15, 16–19]. Degree correlation (μ) is a value that node links to similar or dissimilar node, which was calculated by dividing degree correlation function divided by average number of degree. Modularity (*Mc*) was a fraction of links included within a given group compared to expected links randomly distributed which was calculated using random walks algorithm [22].

### Prognosis prediction

Prognosis of cytokines is analyzed using logistic regression analysis. D0 to D60 single cytokines are entered into the model and only the cytokines that showed statistical significance was entered into the multivariate model. In addition, prediction of survival was analyzed for each cytokines from D0 to D60.

### Statistical analysis

Demographic data and baseline characteristics are presented as median values with ranges for continuous variables. The level of cytokines was compared among D0, D3, D7 and D60 using Kruskal-Wallis test. For molecules with statistical significance, Mann-Whitney test was performed. For the prognosis prediction, univariate and multivariate analyses were performed using Cox regression analysis, and all cytokine molecules were studied by entering the parameters into the model using backward method. Overall survival of patient outcome was analyzed by Kaplan-Meier method and compared by the log rank method. Parameters from multivariate model with statistical significance were used. The starting point of survival was the time of initial diagnosis of hepatocellular carcinoma and primary end point was death from any cause. The maximum area under the ROC curve (AUC) was selected for cut off values of parameters from multivariate analysis. Statistical analyses were performed using Medcalc software version 18.11 (Medcalc, Mariakerke, Belgium).

## Results

### Comparison of D0, D3, D7, and D60 cytokine levels and cytokine correlation analysis

**Fig 1:**
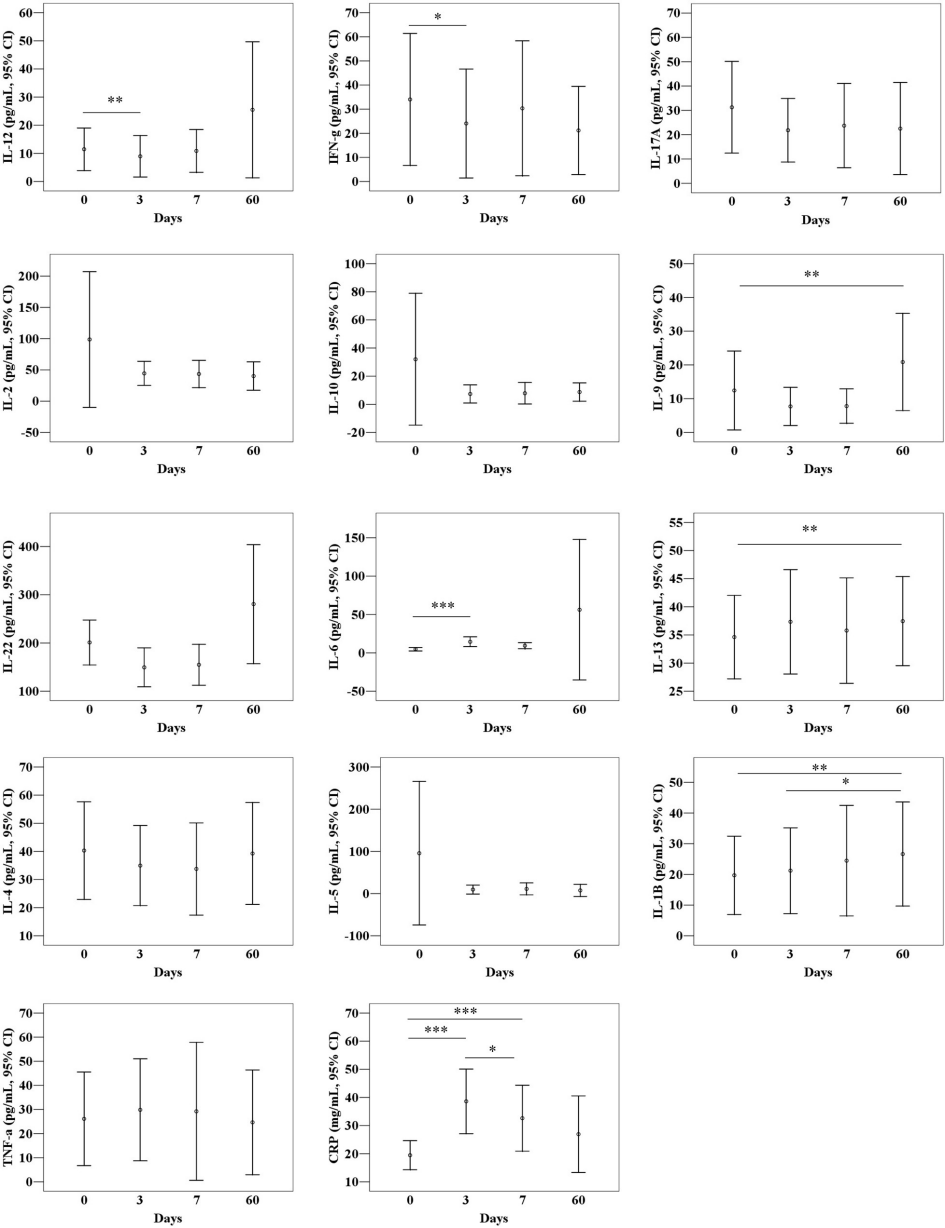
Cytokine concentrations at D0, D3, D7 and D60.

The cytokine profiles on D0, D3, D7 and D60 after TACE showed that CRP level was 19.45, 38.61, 32.62, and 26.94 mg/L, on D0, D3, D7, and D60, respectively, in Table 2 and Fig 1. The IL-6 concentration increased over time from D0 to D60 and peaked at 56.16 ng/mL on D60, a 10-fold increase from the baseline concentration. IL-1β, -9, -12, and IL-22 was increased by D60 compared to that of the baseline concentration. Conversely, IL-2, IL-5, IL-10, IL-17α and IFN-γ concentration decreased over time and was the lowest on D60. IL-4, IL-13, TNF- α concentration was non-fluctuating or steady state on D60.

**Table 2:** Measured cytokine levels for patients before transarterial chemotherapy (D0) and at 3 days (D3), 7 days (D7), and 60 days (D60) post-transarterial chemotherapy^a^.

	D0 (n = 203)		D3 (n = 156)		D7 (n = 147)		D60 (n = 115)	
	Mean	SD	Mean	SD	Mean	SD	Mean	SD
IL-12	11.46	53.95	8.95	46.52	10.86	46.71	25.48	130.98
IFN-γ	34.02	165.42	24.05	142.76	30.33	171.63	21.16	99.06
IL-17α	31.26	134.65	21.81	82.72	23.73	106.26	22.48	102.59
IL-2	98.68	774.16	44.45	122.68	43.53	133.72	40.15	122.75
IL-10	32.01	334.54	7.34	40.74	7.84	46.82	8.719	35.45
IL-9	12.43	83.41	7.71	35.66	7.79	31.23	20.85	77.88
IL-22	201.09	333.01	149.47	255.87	154.84	259.79	280.74	668.21
IL-6	4.77	16.22	14.44	40.36	9.32	24.56	56.16	495.97
IL-13	34.62	52.88	37.33	58.71	35.78	57.49	37.45	42.83
IL-4	40.31	123.77	34.95	89.99	33.75	100.49	39.24	97.95
IL-5	95.61	1212.58	9.56	67.06	11.08	86.32	7.51	76.49
IL-1β	19.70	91.06	21.21	88.35	24.47	110.45	26.62	91.76
TNF-α	26.11	138.56	29.84	133.78	29.22	175.53	24.67	117.66
CRP^a^	19.45	37.14	38.67	41.21	32.62	44.19	26.93	43.72

IL, interleukin; IFN, interferon; TNF, tumor necrosis factor; CRP, C-reactive protein; SD, standard deviation.

^a^Statistical analysis was performed using a Kruskal-Wallis test for analysis of variance among D0, D3,D7and D60 and only CRP revealed statistical significance. Units for cytokines are as follows: CRP, mg/L; others, pg/mL.

Spearman’s correlation method was performed to generate a co-expression matrix in S1–S4 Tables, and the number of molecular pairs significantly correlated at D0, D3, D7, and D60 were as follows: D0, 44 pairs; D3, 58 pairs; D7, 56 pairs; and D60, 50 pairs. These data are listed in S5–S8 Tables.

### Network analysis of molecules

**Fig 2:**
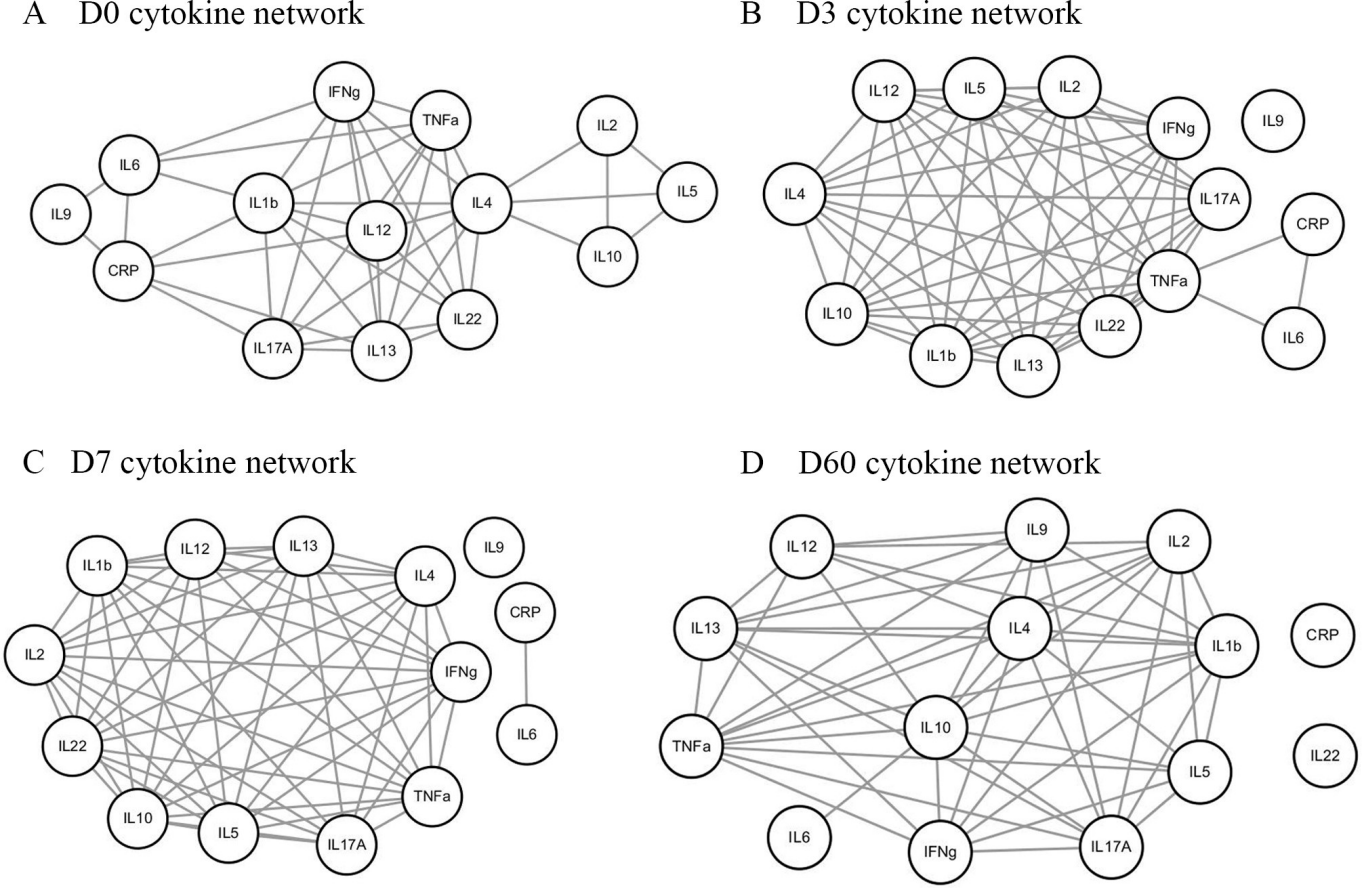
Cytokine Network of hepatocellular carcinoma after TACE. Network analysis results at (A) D0, (B) D3, (C) D7 and (D) D60 after transarterial chemotherapy are presented.

Network was plotted for pairs of cytokines exhibiting significant correlations using correlation coefficients (Fig 2), and topological parameters were calculated (Table 3 and S9–S12 Tables).

**Table 3:** Network topological parameters of D0, D3, D7, and D60 after transarterial chemotherapy.

	D0	D3	D7	D60
Clustering Coefficient, <*C*>	0.797	0.962	0.831	0.813
Network density	0.473	0.731	0.705	0.742
Network heterogeneity	0.398	0.338	0.378	0.307
Connected components	1	1	2	1
Network diameter	4	2	2	2
Network centralization	0.346	0.318	0.152	0.309
Shortest path	182	156	112	132
Characteristic path length	1.681	1.269	1.018	1.258
Average degree, <*k*>	6.143	8.769	8.462	8.167
Number of nodes, *N*	14	13	13	12
Degree correlation, μ	0.281	0.209	1	-0.315
Modularity, *Mc*	0.177	0.038	0.036	-0.091

Analysis of each node revealed that IL-4 had the highest degree (*k* = 10) on D0; TNF-α had the highest degree (*k* = 12) on D3; IL-1β, IL-2, IL-4, IL-5, IL-10, IL-13, IL-22, INF-γ and TNF-α had the highest degree on D7 (*k* = 10); and IL-10 had the highest degree (*k* = 11) on D60 (S9–S12 Tables). The node with the highest degree is considered to be a hub node or, in this study, a hub cytokine. IL-4 was the hub node in D0 network and revealed highest betweenness centrality values, which indicate that removal of IL-4 could disconnect the network. D0, D3, D7, and D60 networks revealed that clustering coefficient, network density, and average degree were highest on D3. These parameters showed similar pattern as CRP. Network heterogeneity and number of included nodes revealed decreasing patterns from D0 to D60, respectively. Modularity (*Mc*) of network revealed constantly decreasing pattern from D0 to D60. Degree correlation (μ) was converted from positive values to negative values by D60. At D0, μ was 0.281, at D3, μ was 0.209 and at D7, μ was 1.00. At D60, μ was -0.315, which implies that the network with assortative nature was turned to disassortative nature by D60 after TACE. Network analysis revealed small network diameter, decreased network heterogeneity, shortest path, characteristic path length by D60 after TACE.

At D0, IL-2, IL-4, IL-5, IL-10 formed a module or clique that seemed to interact intimately each other. At D3, IL-9 was isolated and IL-6 was weakly associated with the network. IL-6, CRP and TNF-α formed a module or clique and that closely interacted each other. At D7, IL-6, IL-9 and CRP were isolated from the network and by D60 IL-22 was isolated. By D60, IL-6 and IL-22 might be playing a role as a regenerative molecule whereas other molecules are playing as a pro- or anti- inflammatory function. Altogether, the network retained an inflammatory function but the cytokine network function seemed to be attenuated by D60.

### Prediction of patient outcome

**Fig 3:**
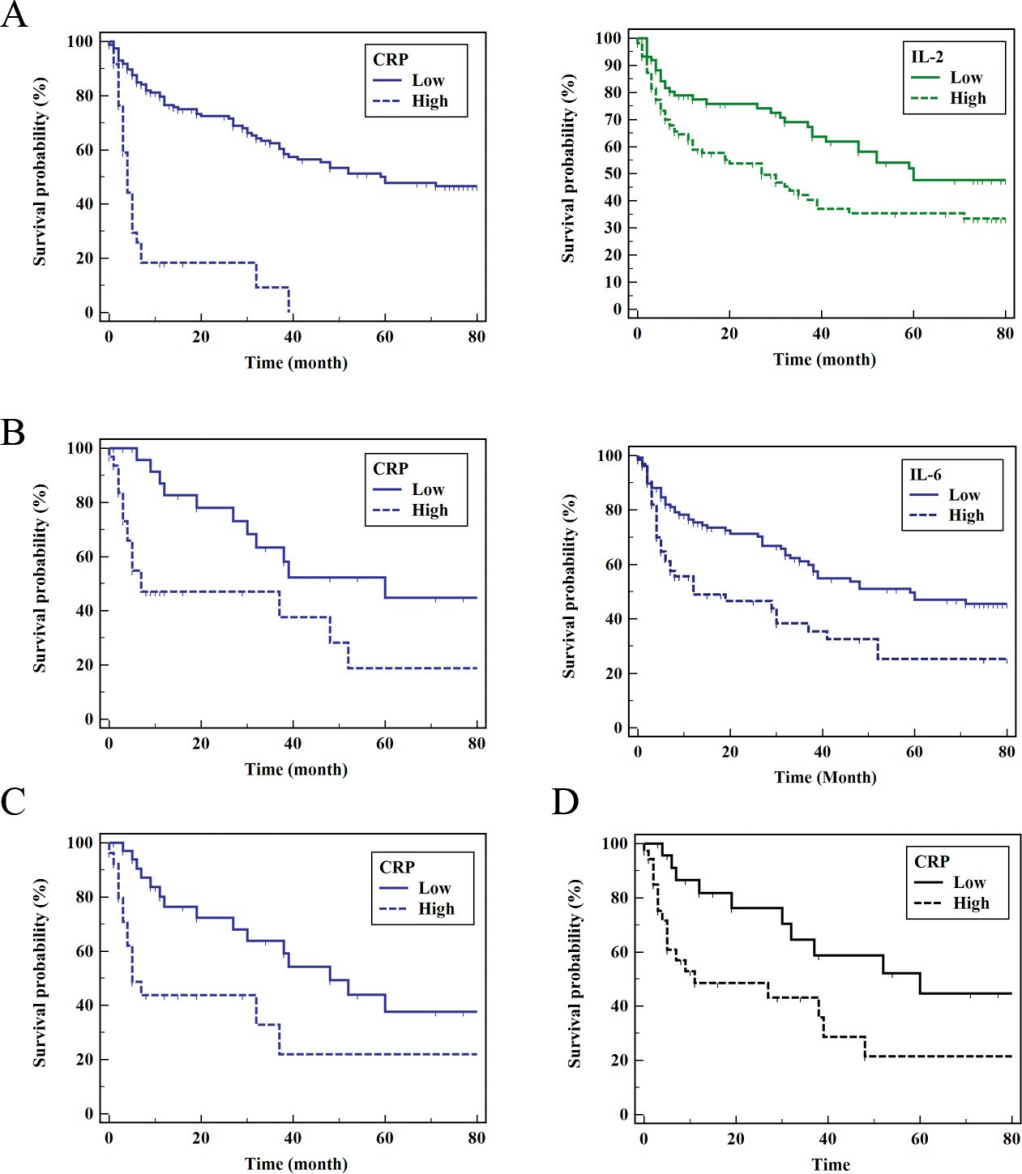
Patient outcome by cytokine status. Overall survival of patient group by high and low concentration of cytokines after transarterial chemotherapy is presented. (A) CRP and IL-2 at D0, (B) CRP and IL-6 at D3,(C) CRP at D7 and (D) CRP at D60.

For prediction of patient outcome, univariate and multivariate analysis was performed using all the studied cytokines from D0, D3 (S13 Table) and D7, D60 (S14 Table). At D0, IL-2, IL-10, IL-6, IL-5, IL-1β, CRP were statistically significant in univariate analysis. In multivariate analysis, IL-2 revealed P-value of 0.012 with a hazard ratio (95% confidence interval, (CI)) of 1.003 (1.001–1.005). CRP revealed P-value of less than 0.001 with a hazard ratio (95% CI) of 1.017 (1.013–1.022). At D3, IL-17α, IL-6, IL-13, CRP revealed statistical significance and in multivariate analysis CRP revealed a P-value of 0.001 with a hazard ratio (95% CI) of 1.015 (1.006–1.024) and IL-6 revealed a P-value of 0.03 and a hazard ratio (95% CI) of 1.012 (1.001–1.022). At D7, only CRP revealed statistical significance in univariate analysis with a P-value of 0.016 with a hazard ratio of 1.009 (1.006–1.017). At D60, IL-12, IL-17α, IL-22, IL-6, IL-1β, TNF-α and CRP revealed statistical significance in univariate analysis. In multivariate analysis only CRP revealed statistical significance with a P-value of less than 0.001 with a hazard ratio (95% CI) of 1.022 (1.010–1.033). ROC analysis revealed that cut off values of D0 CRP was 8.87 mg/L and D0 IL-2 was 1.63 pg/mL. Cut off value of CRP at D3 was 22.39 pg/mL and IL-6 was 3.25 pg/mL. CRP at D7 was 16.27 mg/L and CRP at D60 was 7.53 pg/mL, respectively. Based on these parameters, overall survival was estimated. The five-year survival of hepatocellular carcinoma with TACE patients was studied from the time of diagnosis to death of any cause. Mean survival month of patient group with high and low CRP concentration at D0 was 9.5 and 54.2 months, respectively, with a P-value of less than 0.001 (Fig 3). Of IL-2, patient group with high and low concentration at D0 revealed mean survival month of 39.9 and 56.1 months, respectively, with a P-value of 0.0084. Mean survival month of CRP at D3 was 31.3 and 55.1 months, respectively, with a P-value of 0.0056. IL-6 at D3 revealed mean survival month of 33.7 and 53.1 months for high and low concentration of IL-6 within patient group, respectively with a P-value of 0.0017. At D7, patient group with high and low concentration of CRP revealed 28.7 and 50.7 months, respectively with a P-value of 0.0065. At D60, patient group with high and low concentration of CRP revealed mean survival month of 31.7 and 55.3 months, respectively with a P-value of 0.0135.

## Discussion

Cytokine molecules produced by innate and adaptive immune cells play an important role in the HCC. These local inflammatory responses have systemic effects through direct release of inflammatory cytokines or indirect alteration in inflammatory homeostasis. These systemic effects results in a resetting of the tissue inflammatory state [5–8]. According to the Barcelona Clinic Liver Cancer (BCLC) staging system, patients with intermediate-stage HCC are generally treated with TACE, which reportedly lengthens median survival rates [23–25]. However, TACE often causes inflammation and ischemic injury to the liver, and its effects on systemic inflammation requires elucidation.

Understanding cytokine profiles may provide insight into immunological complexities influenced by treatment-associated inflammation, liver function, and HCC stage [13]. Most of the cytokine profiles revealed an increasing or decreasing pattern. IL-1β, IL-6, IL-9, IL-12, IL-22 level increased and, IL-2, IL-5, IL-10, IL-17α and IFN-γ levels decreased, but IL-4, IL-13, and TNF- α remained stable by D60. Proinflammatory cytokines (IL-1β, IL-12) were increased and anti-inflammatory cytokines (IL-5, IL-10) were decreased by D60. IL-6 and IL-22 seemed to function as a regenerative molecule based on concentration level and network analysis.

IL-6 was correlated with tumor size, number, and metastasis and was an unfavorable prognostic indicator in HCC [13]. Weak association of IL-6 on D7 and D60 indicates that either the expression level of IL-6 was uncontrolled by the cytokine network or negative feedback mechanism of expression of IL-6 was uncontrolled. These might be explained by forming modules with other molecules. IL-6 formed module with CRP from D0 to D7 and related to adverse outcome in D3. These results also indicate that IL-6 was not functioning as a pro- or anti-inflammatory function. IL-6 seemed to be functioning as a regenerative or metastatic molecule. IL-22 is expressed in the liver and related to the proinflammatory function as well as regenerative function [26]. IL-22 stimulates hepatic stem and progenitor cells for regeneration, which was in line with previous results that IL-22 concentration was increased after partial hepatectomy for regenerative response [26, 27]. In this study, IL-22 mediated protection and regeneration from tissue damage in the liver, which was based on concentration level and isolation from the network on D60.

As cytokine profiles are complex, network analysis might better elucidate different aspects of their dynamics that cannot be gleaned via single-cytokine analysis. Network diameter, shortest path, and characteristic path length was related to small world property, which implies that short path length is associated with fast response against external stimuli and adaptation to environmental change [28, 29]. Previous study on protein interaction network revealed that path length was increased in complex organism such as eukaryotes compared to that of prokaryotes [30]. Path length in biologic network was increased to facilitate modularity and the nodes residing in modules have high probability to retain specific function [30, 31]. Modularity in the network was expected to promote evolvability, multifunctionality and robustness of the network [30, 31]. In this study, these parameters were decreased in cytokine networks by D60 after TACE, indicating that the cytokine networks turned out to be relatively efficient in spreading perturbations within the network and in reacting to changes caused by external conditions. In addition, considering that modularity value, diameter, shortest path, characteristic path length were decreased by D60, cytokine network function seemed to be attenuated.

The clustering coefficient and average degree was highest in D3 network and become lower by D60, but the values of these parameters were higher compared to that of baseline values. This pattern was similar to that of CRP concentration, which was the highest in D3. It is unclear whether clustering coefficient and average degree were related to intensity of inflammation in cytokine network. Further studies are required for the relation between these values and CRP.

The degree correlation is a parameter that defines the relation of nodes with other nodes. Assortativeness indicates that a hub node is related to a hub node and dissorativeness indicates a hub node is related to a non-hub node, which is close to hub and spoke model. Degree correlation was decreased by D60 after TACE. These data implies that D60 cytokine network retains scale free feature, which is shown in normal biologic network. Biological networks or scale-free networks tend to exhibit higher network heterogeneity [32, 33]. The systemic inflammation induced by TACE seemed to be attenuate by D60.

A node with the highest degree could be regarded as a hub node, and hub nodes tend to link or interact with nodes with fewer edges [22, 30]. The hub node is related to network robustness, as removal of hub nodes might lead to system failure [32]. In this study, the hubs were IL-4 on D0; TNF-α on D3; IL-1β, IL-2, IL-4, IL-5, IL-10, IL-13, IL-22, INF-γ, and TNF-α on D7; and IFN-γ, IL-2, and IL-10 was the hubs on D60. The IL-4 was the hub in the D0 network. These results imply that IL-4 might play a sophisticated role in immune response among HCC [34]. Removal of the hub increases the probability of disconnection of the network. In addition, IL-4 had the highest betweenness centrality value, which indicates that IL-4 was the bottleneck that connected neighbor nodes. Without IL-4, the probability of disconnection of the network would be increased. Indeed, IL-4 was increased in Child-Turcotte-Pugh class B and C compared to that of class A among HCC patients [34]. In breast cancer, IL-4 was reported to be associated with progression, invasion and tumor growth by downregulating MAPK pathway [35]. Further studies are required for IL-4 and the functional role of this molecule and HCC.

Survival analysis revealed that IL-2 and CRP were associated with unfavorable prognosis in network cytokines at D0. IL-2 and CRP were part of small module within network, which implies the presence of systemic inflammatory response. At D60, only CRP was associated with unfavorable prognosis and CRP was isolated without connection with other cytokines. These results imply that TACE attenuated cytokine network with functional module and turned into network without module.

Limitations of this study are that the etiology of HCC in this study was mostly due to hepatitis B virus, which might have a different systemic inflammatory response compared to other causes and the patients were predominantly male, which might have affected the outcome. In addition, the cytokine network might have been better analyzed using a time series algorithm, which was mostly designed for analysis of gene expression data. Comparisons of network topological parameters using Z scores were statistically impossible due to the use of four networks.

## Conclusion

Network diameter, centralization, modularity, degree correlation, characteristic path length and shortest path length after 60 days of TACE seemed that the cytokine network function was attenuated. Although cytokine network retained inflammatory function, these network parameters indicated that cytokine network function was attenuated by D60. At D0 network, IL-4, IL-5, IL-10 and IL-2 formed a module and IL-4 was the hub node at D0. On D3, network revealed the highest clustering coefficient and the average degree that was similar with CRP concentration. These network parameters might be related to the inflammation intensity within cytokine network. On D7, IL-6 and IL-9 were isolated from the network, which seemed to have regenerative function and anti-tumor effect, respectively. On, D60, IL-6 and IL-22 were weakly related to or isolated from the network, respectively. IL-6 and IL-22 seemed to play a regenerative function but not pro- or anti- inflammatory function.

## Supporting information

S1 Table Correlation matrix of cytokines concentrations at D0. (DOCX)

S2 Table Correlation matrix of cytokines concentrations at D3. (DOCX)

S3 Table Correlation matrix of cytokines concentrations at D7. (DOCX)

S4 Table Correlation matrix of cytokines concentrations at D60. (DOCX)

S5 Table P-value of correlation matrix from cytokines concentrations at D0. (DOCX)

S6 Table P-value of correlation matrix from cytokines concentrations at D3. (DOCX)

S7 Table P-value of correlation matrix from cytokines concentrations at D7. (DOCX)

S8 Table P-value of correlation matrix from cytokines concentrations at D60. (DOCX)

S9 Table Topological parameters from network analysis of D0. (DOCX)

S10 Table Topological parameters from network analysis of D3. (DOCX)

S11 Table Topological parameters from network analysis of D7. (DOCX)

S12 Table Topological parameters from network analysis of D60. (DOCX)

S13 Table Univariate and multivariate analysis by Cox regression analysis for D0 and D3. (DOCX)

S14 Table Univariate and multivariate analysis by Cox regression analysis for D7 and D60. (DOCX)
