# Correction: Complex interaction networks of cytokines after transarterial chemotherapy in patients with hepatocellular carcinoma

**DOI:** 10.1371/journal.pone.0229552

**Published:** 2020-02-19

**Authors:** Dong Wook Jekarl, Seungok Lee, Jung Hyun Kwon, Soon Woo Nam, Myungshin Kim, Yonggoo Kim, Jeong Won Jang

The Funding statement is incorrect. The correct Funding statement is as follows: This research was supported by a Grant of Translational R&D Project through Institute for Bio-Medical convergence, Incheon St. Mary's Hospital, The Catholic University of Korea. This study was supported by Basic Science Research Program through the National Research Foundation of Korea (NRF) funded by the Ministry of Science, ICT & Future Planning (NRF-2019R1A2C1009439).
