# Supplementary material for: Complex interaction networks of cytokines after transarterial chemotherapy in patients with hepatocellular carcinoma
Source: PLoS One. 2019 Nov 21;14(11):e0224318. doi: 10.1371/journal.pone.0224318 (PMC6874208; doi:10.1371/journal.pone.0224318)
Supplement: S1 Table — (DOCX) [file pone.0224318.s001.docx]

S1 Table. Correlation matrix of cytokines concentrations at D0.

|  | IL-12p70 | IFN-γ | IL-17α | IL-2 | IL-10 | IL-9 | IL-22 | IL-6 | IL-13 | IL-4 | IL-5 | IL-1β | TNF-α | CRP |
| --- | --- | --- | --- | --- | --- | --- | --- | --- | --- | --- | --- | --- | --- | --- |
| IL-12p70 | 1 | 0.54 | 0.49 | 0.2 | 0.39 | 0.3 | 0.26 | 0.2 | 0.24 | 0.42 | 0.4 | 0.44 | 0.38 | -0.01 |
| IFN-γ | 0.54 | 1 | 0.57 | 0.24 | 0.32 | 0.3 | 0.19 | 0.18 | 0.26 | 0.43 | 0.34 | 0.33 | 0.33 | -0.01 |
| IL-17α | 0.49 | 0.57 | 1 | 0.12 | 0.32 | 0.2 | 0.21 | 0.01 | 0.26 | 0.34 | 0.36 | 0.42 | 0.22 | 0.01 |
| IL-2 | 0.2 | 0.24 | 0.12 | 1 | 0.4 | 0.1 | 0.4 | 0.01 | 0.34 | 0.34 | 0.37 | 0.3 | 0.29 | 0.16 |
| IL-10 | 0.39 | 0.32 | 0.32 | 0.4 | 1 | 0.16 | 0.4 | 0.13 | 0.21 | 0.5 | 0.38 | 0.33 | 0.42 | 0.07 |
| IL-9 | 0.3 | 0.3 | 0.2 | 0.1 | 0.16 | 1 | 0.11 | 0.15 | 0.15 | 0.25 | 0.13 | 0.19 | 0.19 | 0.02 |
| IL-22 | 0.26 | 0.19 | 0.21 | 0.4 | 0.4 | 0.11 | 1 | -0.03 | 0.04 | 0.27 | 0.2 | 0.36 | 0.19 | -0.04 |
| IL-6 | 0.2 | 0.18 | 0.01 | 0.01 | 0.13 | 0.15 | -0.03 | 1 | 0.02 | 0.1 | 0.22 | 0.16 | 0.2 | 0.34 |
| IL-13 | 0.24 | 0.26 | 0.26 | 0.34 | 0.21 | 0.15 | 0.04 | 0.02 | 1 | 0.29 | 0.29 | 0.35 | 0.32 | 0 |
| IL-4 | 0.42 | 0.43 | 0.34 | 0.34 | 0.5 | 0.25 | 0.27 | 0.1 | 0.29 | 1 | 0.42 | 0.39 | 0.5 | 0.03 |
| IL-5 | 0.4 | 0.34 | 0.36 | 0.37 | 0.38 | 0.13 | 0.2 | 0.22 | 0.29 | 0.42 | 1 | 0.23 | 0.32 | 0.09 |
| IL-1β | 0.44 | 0.33 | 0.42 | 0.3 | 0.33 | 0.19 | 0.36 | 0.16 | 0.35 | 0.39 | 0.23 | 1 | 0.37 | 0.03 |
| TNF-α | 0.38 | 0.33 | 0.22 | 0.29 | 0.42 | 0.19 | 0.19 | 0.2 | 0.32 | 0.5 | 0.32 | 0.37 | 1 | 0.18 |
| CRP | -0.01 | -0.01 | 0.01 | 0.16 | 0.07 | 0.02 | -0.04 | 0.34 | 0 | 0.03 | 0.09 | 0.03 | 0.18 | 1 |

IL, interleukin; IFN, interferon; TNF, tumor necrosis factor; CRP, C-reactive protein
