# Supplementary material for: Complex interaction networks of cytokines after transarterial chemotherapy in patients with hepatocellular carcinoma
Source: PLoS One. 2019 Nov 21;14(11):e0224318. doi: 10.1371/journal.pone.0224318 (PMC6874208; doi:10.1371/journal.pone.0224318)
Supplement: S2 Table — (DOCX) [file pone.0224318.s002.docx]

S2 Table. Correlation matrix of cytokines concentrations at D3

|  | IL-12p70 | IFN-γ | IL-17α | IL-2 | IL-10 | IL-9 | IL-22 | IL-6 | IL-13 | IL-4 | IL-5 | IL-1β | TNF-α | CRP |
| --- | --- | --- | --- | --- | --- | --- | --- | --- | --- | --- | --- | --- | --- | --- |
| IL-12p70 | 1 | 0.66 | 0.59 | 0.29 | 0.51 | 0.24 | 0.35 | 0.2 | 0.38 | 0.48 | 0.45 | 0.39 | 0.54 | -0.16 |
| IFN-γ | 0.66 | 1 | 0.61 | 0.18 | 0.4 | 0.24 | 0.24 | 0.18 | 0.35 | 0.54 | 0.49 | 0.33 | 0.44 | -0.13 |
| IL-17α | 0.59 | 0.61 | 1 | 0.14 | 0.34 | 0.18 | 0.13 | 0.11 | 0.29 | 0.51 | 0.49 | 0.41 | 0.4 | -0.4 |
| IL-2 | 0.29 | 0.18 | 0.14 | 1 | 0.33 | 0.04 | 0.22 | 0.04 | 0.31 | 0.27 | 0.39 | 0.25 | 0.25 | -0.06 |
| IL-10 | 0.51 | 0.4 | 0.34 | 0.33 | 1 | 0.16 | 0.25 | 0.1 | 0.19 | 0.28 | 0.37 | 0.36 | 0.42 | -0.06 |
| IL-9 | 0.24 | 0.24 | 0.18 | 0.04 | 0.16 | 1 | 0.04 | 0.02 | 0.24 | 0.25 | 0.16 | 0.14 | 0.1 | -0.07 |
| IL-22 | 0.35 | 0.24 | 0.13 | 0.22 | 0.25 | 0.04 | 1 | 0.08 | 0.26 | 0.14 | 0.25 | 0.26 | 0.23 | 0.06 |
| IL-6 | 0.2 | 0.18 | 0.11 | 0.04 | 0.1 | 0.02 | 0.08 | 1 | 0.14 | 0 | 0.08 | 0.2 | 0.12 | 0.4 |
| IL-13 | 0.38 | 0.35 | 0.29 | 0.31 | 0.19 | 0.24 | 0.26 | 0.14 | 1 | 0.22 | 0.41 | 0.37 | 0.33 | 0.11 |
| IL-4 | 0.48 | 0.54 | 0.51 | 0.27 | 0.28 | 0.25 | 0.14 | 0 | 0.22 | 1 | 0.39 | 0.25 | 0.36 | -0.1 |
| IL-5 | 0.45 | 0.49 | 0.49 | 0.39 | 0.37 | 0.16 | 0.25 | 0.08 | 0.41 | 0.39 | 1 | 0.34 | 0.41 | -0.1 |
| IL-1β | 0.39 | 0.33 | 0.41 | 0.25 | 0.36 | 0.14 | 0.26 | 0.2 | 0.37 | 0.25 | 0.34 | 1 | 0.58 | -0.05 |
| TNF-α | 0.54 | 0.44 | 0.4 | 0.25 | 0.42 | 0.1 | 0.23 | 0.12 | 0.33 | 0.36 | 0.41 | 0.58 | 1 | 0.11 |
| CRP | -0.16 | -0.13 | -0.4 | -0.06 | -0.06 | -0.07 | 0.06 | 0.4 | 0.11 | -0.1 | -0.1 | -0.05 | 0.11 | 1 |

IL, interleukin; IFN, interferon; TNF, tumor necrosis factor; CRP, C-reactive protein
