# Supplementary material for: Complex interaction networks of cytokines after transarterial chemotherapy in patients with hepatocellular carcinoma
Source: PLoS One. 2019 Nov 21;14(11):e0224318. doi: 10.1371/journal.pone.0224318 (PMC6874208; doi:10.1371/journal.pone.0224318)
Supplement: S5 Table — (DOCX) [file pone.0224318.s005.docx]

S5 Table. P-value of correlation matrix from cytokines concentrations at D0

|  | IL-12p70 | IFN-γ | IL-17α | IL-2 | IL-10 | IL-9 | IL-22 | IL-6 | IL-13 | IL-4 | IL-5 | IL-1β | TNF-α | CRP |
| --- | --- | --- | --- | --- | --- | --- | --- | --- | --- | --- | --- | --- | --- | --- |
| IL-12p70 | 0 | 4.44E-16 | 2.49E-13 | 0.0046892 | 8.82E-09 | 2.19E-05 | 0.0002572 | 0.0043542 | 0.0007919 | 7.83E-10 | 5.58E-09 | 1.07E-10 | 2.35E-08 | 0.8802453 |
| IFN-γ | 4.44E-16 | 0 | 0 | 0.000859 | 5.03E-06 | 2.07E-05 | 0.0077902 | 0.0098588 | 0.0001939 | 2.97E-10 | 7.36E-07 | 2.18E-06 | 1.49E-06 | 0.9383169 |
| IL-17α | 2.49E-13 | 0 | 0 | 0.0984819 | 3.71E-06 | 0.0042205 | 0.0028288 | 0.8760488 | 0.0002485 | 1.37E-06 | 1.51E-07 | 8.11E-10 | 0.0015132 | 0.8751653 |
| IL-2 | 0.0046892 | 0.000859 | 0.0984819 | 0 | 3.86E-09 | 0.1638558 | 4.19E-09 | 0.8873385 | 1.18E-06 | 1.06E-06 | 9.11E-08 | 2.09E-05 | 3.78E-05 | 0.0212767 |
| IL-10 | 8.82E-09 | 5.03E-06 | 3.71E-06 | 3.86E-09 | 0 | 0.0282777 | 4.01E-09 | 0.0653749 | 0.0024227 | 7.68E-14 | 2.49E-08 | 2.69E-06 | 9.51E-10 | 0.3259217 |
| IL-9 | 2.19E-05 | 2.07E-05 | 0.0042205 | 0.1638558 | 0.0282777 | 0 | 0.1322872 | 0.0329701 | 0.0403759 | 0.0004201 | 0.0784509 | 0.0075907 | 0.0088953 | 0.7308622 |
| IL-22 | 0.0002572 | 0.0077902 | 0.0028288 | 4.19E-09 | 4.01E-09 | 0.1322872 | 0 | 0.6872993 | 0.6144581 | 9.24E-05 | 0.0040382 | 2.35E-07 | 0.0077195 | 0.5535554 |
| IL-6 | 0.0043542 | 0.0098588 | 0.8760488 | 0.8873385 | 0.0653749 | 0.0329701 | 0.6872993 | 0 | 0.7868484 | 0.1764893 | 0.0014525 | 0.0240984 | 0.0043721 | 1.15E-06 |
| IL-13 | 0.0007919 | 0.0001939 | 0.0002485 | 1.18E-06 | 0.0024227 | 0.0403759 | 0.6144581 | 0.7868484 | 0 | 2.74E-05 | 4.31E-05 | 3.13E-07 | 6.09E-06 | 1.00E+00 |
| IL-4 | 7.83E-10 | 2.97E-10 | 1.37E-06 | 1.06E-06 | 7.68E-14 | 0.0004201 | 9.24E-05 | 0.1764893 | 2.74E-05 | 0 | 5.28E-10 | 1.15E-08 | 1.17E-13 | 0.7063812 |
| IL-5 | 5.58E-09 | 7.36E-07 | 1.51E-07 | 9.11E-08 | 2.49E-08 | 0.0784509 | 0.0040382 | 0.0014525 | 4.31E-05 | 5.28E-10 | 0 | 0.0011427 | 4.60E-06 | 0.2017491 |
| IL-1β | 1.07E-10 | 2.18E-06 | 8.11E-10 | 2.09E-05 | 2.69E-06 | 0.0075907 | 2.35E-07 | 0.0240984 | 3.13E-07 | 1.15E-08 | 0.0011427 | 0 | 7.19E-08 | 0.6354956 |
| TNF-α | 2.35E-08 | 1.49E-06 | 0.0015132 | 3.78E-05 | 9.51E-10 | 0.0088953 | 0.0077195 | 0.0043721 | 6.09E-06 | 1.17E-13 | 4.60E-06 | 7.19E-08 | 0 | 0.0120675 |
| CRP | 0.8802453 | 0.9383169 | 0.8751653 | 0.0212767 | 0.3259217 | 0.7308622 | 0.5535554 | 1.15E-06 | 1.00E+00 | 0.7063812 | 0.2017491 | 0.6354956 | 0.0120675 | 0 |

IL, interleukin; IFN, interferon; TNF, tumor necrosis factor; CRP, C-reactive protein
