# Supplementary material for: Complex interaction networks of cytokines after transarterial chemotherapy in patients with hepatocellular carcinoma
Source: PLoS One. 2019 Nov 21;14(11):e0224318. doi: 10.1371/journal.pone.0224318 (PMC6874208; doi:10.1371/journal.pone.0224318)
Supplement: S6 Table — (DOCX) [file pone.0224318.s006.docx]

S6 Table.P-value of correlation matrix from cytokines concentrations at D3

|  | IL-12p70 | IFN-γ | IL-17α | IL-2 | IL-10 | IL-9 | IL-22 | IL-6 | IL-13 | IL-4 | IL-5 | IL-1β | TNF-α | CRP |
| --- | --- | --- | --- | --- | --- | --- | --- | --- | --- | --- | --- | --- | --- | --- |
| IL-12p70 | 0 | 0 | 4.44E-16 | 0.000203 | 1.29E-11 | 0.002289 | 1.02E-05 | 0.012649 | 7.44E-07 | 2.17E-10 | 3.32E-09 | 4.56E-07 | 2.56E-13 | 0.279274 |
| IFN-γ | 0 | 0 | 0 | 0.025793 | 2.18E-07 | 0.002723 | 0.002412 | 0.026874 | 6.50E-06 | 4.85E-13 | 5.38E-11 | 2.45E-05 | 6.15E-09 | 0.411636 |
| IL-17α | 4.44E-16 | 0 | 0 | 0.082331 | 1.13E-05 | 0.026372 | 0.09597 | 0.172501 | 0.000199 | 1.76E-11 | 6.84E-11 | 1.20E-07 | 2.53E-07 | 0.006828 |
| IL-2 | 0.0002029 | 0.025793 | 0.082331 | 0 | 2.95E-05 | 0.60879 | 0.006066 | 0.611784 | 9.45E-05 | 0.000531 | 5.51E-07 | 0.001691 | 0.001879 | 0.699338 |
| IL-10 | 1.29E-11 | 2.18E-07 | 1.13E-05 | 2.95E-05 | 0 | 0.052987 | 0.001521 | 0.200121 | 0.018809 | 0.000498 | 2.47E-06 | 3.15E-06 | 5.33E-08 | 0.680972 |
| IL-9 | 0.0022892 | 0.002723 | 0.026372 | 0.60879 | 0.052987 | 0 | 0.6057 | 0.757681 | 0.002999 | 0.001547 | 0.042261 | 0.088541 | 0.228333 | 0.659237 |
| IL-22 | 1.02E-05 | 0.002412 | 0.09597 | 0.006066 | 0.001521 | 0.6057 | 0 | 0.298339 | 0.001238 | 0.074393 | 0.001556 | 0.000895 | 0.003893 | 0.69333 |
| IL-6 | 0.012649 | 0.026874 | 0.172501 | 0.611784 | 0.200121 | 0.757681 | 0.298339 | 0 | 0.077826 | 0.986968 | 0.320232 | 0.012644 | 0.120147 | 0.006006 |
| IL-13 | 7.44E-07 | 6.50E-06 | 0.000199 | 9.45E-05 | 0.018809 | 0.002999 | 0.001238 | 0.077826 | 0 | 0.006008 | 9.36E-08 | 2.79E-06 | 2.22E-05 | 0.452478 |
| IL-4 | 2.17E-10 | 4.85E-13 | 1.76E-11 | 0.000531 | 0.000498 | 0.001547 | 0.074393 | 0.986968 | 0.006008 | 0 | 4.60E-07 | 0.00167 | 2.92E-06 | 0.502843 |
| IL-5 | 3.32E-09 | 5.38E-11 | 6.84E-11 | 5.51E-07 | 2.47E-06 | 0.042261 | 0.001556 | 0.320232 | 9.36E-08 | 4.60E-07 | 0 | 1.30E-05 | 7.42E-08 | 0.494624 |
| IL-1β | 4.56E-07 | 2.45E-05 | 1.20E-07 | 0.001691 | 3.15E-06 | 0.088541 | 0.000895 | 0.012644 | 2.79E-06 | 0.00167 | 1.30E-05 | 0 | 1.33E-15 | 0.742564 |
| TNF-α | 2.56E-13 | 6.15E-09 | 2.53E-07 | 0.001879 | 5.33E-08 | 0.228333 | 0.003893 | 0.120147 | 2.22E-05 | 2.92E-06 | 7.42E-08 | 1.33E-15 | 0 | 0.479021 |
| CRP | 0.2792745 | 0.411636 | 0.006828 | 0.699338 | 0.680972 | 0.659237 | 0.69333 | 0.006006 | 0.452478 | 0.502843 | 0.494624 | 0.742564 | 0.479021 | 0 |

IL, interleukin; IFN, interferon; TNF, tumor necrosis factor; CRP, C-reactive protein
