# Supplementary material for: Complex interaction networks of cytokines after transarterial chemotherapy in patients with hepatocellular carcinoma
Source: PLoS One. 2019 Nov 21;14(11):e0224318. doi: 10.1371/journal.pone.0224318 (PMC6874208; doi:10.1371/journal.pone.0224318)
Supplement: S7 Table — (DOCX) [file pone.0224318.s007.docx]

S7 Table. P-value of correlation matrix from cytokines concentrationsat D7

|  | IL-12p70 | IFN-γ | IL-17α | IL-2 | IL-10 | IL-9 | IL-22 | IL-6 | IL-13 | IL-4 | IL-5 | IL-1β | TNF-α | CRP |
| --- | --- | --- | --- | --- | --- | --- | --- | --- | --- | --- | --- | --- | --- | --- |
| IL-12p70 | NA | 2.20E-12 | 1.05E-08 | 0.0476501 | 3.55E-04 | 2.36E-05 | 0.0013362 | 0.431653 | 3.27E-02 | 8.44E-05 | 3.28E-03 | 9.25E-04 | 2.58E-09 | 0.9355693 |
| IFN-γ | 2.20E-12 | NA | 0.00E+00 | 0.2520971 | 4.24E-03 | 0.6397112 | 1.26E-05 | 0.0587476 | 0.0940296 | 1.29E-04 | 0.0170626 | 1.51E-03 | 7.78E-06 | 0.7682581 |
| IL-17α | 1.05E-08 | 0.00E+00 | NA | 0.3601983 | 1.65E-04 | 0.2089215 | 0.0010735 | 0.2149995 | 0.2053476 | 2.76E-03 | 1.71E-02 | 2.90E-02 | 7.29E-04 | 0.4921731 |
| IL-2 | 0.0476501 | 0.2520971 | 0.3601983 | NA | 0.00715 | 0.0307892 | 1.26E-01 | 0.2929361 | 5.22E-04 | 9.68E-01 | 0.0289788 | 0.1382228 | 0.1133881 | 0.5705176 |
| IL-10 | 3.55E-04 | 4.24E-03 | 1.65E-04 | 0.00715 | NA | 0.1504151 | 2.65E-04 | 0.012208 | 0.2123983 | 5.45E-02 | 0.0027096 | 2.59E-09 | 1.57E-08 | 0.9973282 |
| IL-9 | 2.36E-05 | 0.6397112 | 0.2089215 | 0.0307892 | 0.1504151 | NA | 0.0274385 | 0.2632603 | 0.9935945 | 0.3508366 | 0.7157218 | 0.2375944 | 0.8099609 | 0.9420941 |
| IL-22 | 0.0013362 | 1.26E-05 | 0.0010735 | 1.26E-01 | 2.65E-04 | 0.0274385 | NA | 0.0050372 | 3.99E-01 | 0.0277082 | 0.1477419 | 0.0002253 | 0.0007398 | 0.6452936 |
| IL-6 | 0.431653 | 0.0587476 | 0.2149995 | 0.2929361 | 0.012208 | 0.2632603 | 0.0050372 | NA | 0.2354991 | 0.1454391 | 0.9915599 | 0.0056409 | 0.0063392 | 0.0180338 |
| IL-13 | 3.27E-02 | 0.0940296 | 0.2053476 | 5.22E-04 | 0.2123983 | 0.9935945 | 3.99E-01 | 0.2354991 | NA | 0.0001731 | 0.3144494 | 0.1073154 | 0.3283636 | 0.1630269 |
| IL-4 | 8.44E-05 | 1.29E-04 | 2.76E-03 | 9.68E-01 | 5.45E-02 | 0.3508366 | 0.0277082 | 0.1454391 | 0.0001731 | NA | 2.96E-02 | 3.17E-06 | 3.90E-10 | 0.4185415 |
| IL-5 | 3.28E-03 | 0.0170626 | 1.71E-02 | 0.0289788 | 0.0027096 | 0.7157218 | 0.1477419 | 0.9915599 | 0.3144494 | 2.96E-02 | NA | 0.6686225 | 1.81E-02 | 0 |
| IL-1β | 9.25E-04 | 1.51E-03 | 2.90E-02 | 0.1382228 | 2.59E-09 | 0.2375944 | 0.0002253 | 0.0056409 | 0.1073154 | 3.17E-06 | 0.6686225 | NA | 2.50E-13 | 0.493075 |
| TNF-α | 2.58E-09 | 7.78E-06 | 7.29E-04 | 0.1133881 | 1.57E-08 | 0.8099609 | 0.0007398 | 0.0063392 | 0.3283636 | 3.90E-10 | 1.81E-02 | 2.50E-13 | NA | 0.7538161 |
| CRP | 0.9355693 | 0.7682581 | 0.4921731 | 0.5705176 | 0.9973282 | 0.9420941 | 0.6452936 | 0.0180338 | 0.1630269 | 0.4185415 | 0 | 0.493075 | 0.7538161 | NA |

IL, interleukin; IFN, interferon; TNF, tumor necrosis factor; CRP, C-reactive protein
