# Supplementary material for: Complex interaction networks of cytokines after transarterial chemotherapy in patients with hepatocellular carcinoma
Source: PLoS One. 2019 Nov 21;14(11):e0224318. doi: 10.1371/journal.pone.0224318 (PMC6874208; doi:10.1371/journal.pone.0224318)
Supplement: S8 Table — (DOCX) [file pone.0224318.s008.docx]

S8 Table. P-value of correlation matrix from cytokines concentrationsat D60

|  | IL-12p70 | IFN-γ | IL-17α | IL-2 | IL-10 | IL-9 | IL-22 | IL-6 | IL-13 | IL-4 | IL-5 | IL-1β | TNF-α | CRP |
| --- | --- | --- | --- | --- | --- | --- | --- | --- | --- | --- | --- | --- | --- | --- |
| IL-12p70 | NA | 2.20E-12 | 1.05E-08 | 0.04765 | 0.000355 | 2.36E-05 | 0.001336 | 0.431653 | 0.032662 | 8.44E-05 | 0.003285 | 0.000925 | 2.58E-09 | 0.935569 |
| IFN-γ | 2.20E-12 | NA | 0 | 0.252097 | 0.004239 | 0.639711 | 1.26E-05 | 0.058748 | 0.09403 | 0.000129 | 0.017063 | 0.001508 | 7.78E-06 | 0.768258 |
| IL-17α | 1.05E-08 | 0 | NA | 0.360198 | 0.000165 | 0.208922 | 0.001074 | 0.214999 | 0.205348 | 0.002761 | 0.017063 | 0.028966 | 0.000729 | 0.492173 |
| IL-2 | 0.04765 | 0.252097 | 0.360198 | NA | 0.00715 | 0.030789 | 0.12563 | 0.292936 | 0.000522 | 0.968419 | 0.028979 | 0.138223 | 0.113388 | 0.570518 |
| IL-10 | 0.000355 | 0.004239 | 0.000165 | 0.00715 | NA | 0.150415 | 0.000265 | 0.012208 | 0.212398 | 0.054542 | 0.00271 | 2.59E-09 | 1.57E-08 | 0.997328 |
| IL-9 | 2.36E-05 | 0.639711 | 0.208922 | 0.030789 | 0.150415 | NA | 0.027439 | 0.26326 | 0.993594 | 0.350837 | 0.715722 | 0.237594 | 0.809961 | 0.942094 |
| IL-22 | 0.001336 | 1.26E-05 | 0.001074 | 0.12563 | 0.000265 | 0.027439 | NA | 0.005037 | 0.399265 | 0.027708 | 0.147742 | 0.000225 | 0.00074 | 0.645294 |
| IL-6 | 0.431653 | 0.058748 | 0.214999 | 0.292936 | 0.012208 | 0.26326 | 0.005037 | NA | 0.235499 | 0.145439 | 0.99156 | 0.005641 | 0.006339 | 0.018034 |
| IL-13 | 0.032662 | 0.09403 | 0.205348 | 0.000522 | 0.212398 | 0.993594 | 0.399265 | 0.235499 | NA | 0.000173 | 0.314449 | 0.107315 | 0.328364 | 0.163027 |
| IL-4 | 8.44E-05 | 0.000129 | 0.002761 | 0.968419 | 0.054542 | 0.350837 | 0.027708 | 0.145439 | 0.000173 | NA | 0.029591 | 3.17E-06 | 3.90E-10 | 0.418542 |
| IL-5 | 0.003285 | 0.017063 | 0.017063 | 0.028979 | 0.00271 | 0.715722 | 0.147742 | 0.99156 | 0.314449 | 0.029591 | NA | 0.668622 | 0.018052 | NA |
| IL-1β | 0.000925 | 0.001508 | 0.028966 | 0.138223 | 2.59E-09 | 0.237594 | 0.000225 | 0.005641 | 0.107315 | 3.17E-06 | 0.668622 | NA | 2.50E-13 | 0.493075 |
| TNF-α | 2.58E-09 | 7.78E-06 | 0.000729 | 0.113388 | 1.57E-08 | 0.809961 | 0.00074 | 0.006339 | 0.328364 | 3.90E-10 | 0.018052 | 2.50E-13 | NA | 0.753816 |
| CRP | 0.935569 | 0.768258 | 0.492173 | 0.570518 | 0.997328 | 0.942094 | 0.645294 | 0.018034 | 0.163027 | 0.418542 | NA | 0.493075 | 0.753816 | NA |

IL, interleukin; IFN, interferon; TNF, tumor necrosis factor; CRP, C-reactive protein
