# Supplementary material for: Complex interaction networks of cytokines after transarterial chemotherapy in patients with hepatocellular carcinoma
Source: PLoS One. 2019 Nov 21;14(11):e0224318. doi: 10.1371/journal.pone.0224318 (PMC6874208; doi:10.1371/journal.pone.0224318)
Supplement: S9 Table — (DOCX) [file pone.0224318.s009.docx]

S9 Table. Topological parameters from network analysis of D0

|  | Average shortest | Clustering | Closeness | Stress | Degree | Betweenness | Neighborhood | Topological |
| --- | --- | --- | --- | --- | --- | --- | --- | --- |
|  | path length | coefficient | centrality |  |  | centrality | connectivity | coefficient |
| CRP | 1.769 | 0.467 | 0.565 | 56 | 6 | 0.093 | 6.333 | 0.633 |
| IFN-γ | 1.385 | 0.786 | 0.722 | 28 | 8 | 0.042 | 7.625 | 0.587 |
| IL-10 | 2.077 | 1.000 | 0.481 | 0 | 3 | 0.000 | 5.333 | 0.533 |
| IL-12 | 1.462 | 0.810 | 0.684 | 24 | 7 | 0.030 | 8.000 | 0.615 |
| IL-13 | 1.385 | 0.821 | 0.722 | 26 | 8 | 0.032 | 7.750 | 0.596 |
| IL-17α | 1.462 | 0.810 | 0.684 | 24 | 7 | 0.030 | 8.000 | 0.615 |
| IL-1β | 1.308 | 0.722 | 0.765 | 52 | 9 | 0.072 | 7.333 | 0.564 |
| IL-2 | 2.077 | 1.000 | 0.481 | 0 | 3 | 0.000 | 5.333 | 0.533 |
| IL-22 | 1.538 | 0.952 | 0.650 | 2 | 7 | 0.002 | 8.143 | 0.679 |
| IL-4 | 1.308 | 0.511 | 0.765 | 128 | 10 | 0.386 | 6.300 | 0.525 |
| IL-5 | 2.077 | 1.000 | 0.481 | 0 | 3 | 0.000 | 5.333 | 0.533 |
| IL-6 | 1.846 | 0.500 | 0.542 | 40 | 5 | 0.065 | 6.600 | 0.660 |
| IL-9 | 2.462 | 1.000 | 0.406 | 0 | 2 | 0.000 | 5.500 | 0.688 |
| TNF-α | 1.385 | 0.786 | 0.722 | 28 | 8 | 0.042 | 7.625 | 0.587 |

IL, interleukin; IFN, interferon; TNF, tumor necrosis factor; CRP, C-reactive protein
