# Supplementary material for: Complex interaction networks of cytokines after transarterial chemotherapy in patients with hepatocellular carcinoma
Source: PLoS One. 2019 Nov 21;14(11):e0224318. doi: 10.1371/journal.pone.0224318 (PMC6874208; doi:10.1371/journal.pone.0224318)
Supplement: S11 Table — (DOCX) [file pone.0224318.s011.docx]

S11 Table. Topological parameters from network analysis of D7

|  | Average shortest | Clustering | Closeness | Stress | Degree | Betweenness | Neighborhood | Topological |
| --- | --- | --- | --- | --- | --- | --- | --- | --- |
|  | path length | coefficient | centrality |  |  | centrality | connectivity | coefficient |
| CRP | 1.000 | 0.000 | 1.000 | 0 | 1 | 0.000 | 1.000 | 0.000 |
| IFN-γ | 1.000 | 0.978 | 1.000 | 2 | 10 | 0.002 | 9.800 | 0.980 |
| IL-10 | 1.000 | 0.978 | 1.000 | 2 | 10 | 0.002 | 9.800 | 0.980 |
| IL-12 | 1.100 | 1.000 | 0.909 | 0 | 9 | 0.000 | 10.000 | 1.000 |
| IL-13 | 1.000 | 0.978 | 1.000 | 2 | 10 | 0.002 | 9.800 | 0.980 |
| IL-17α | 1.100 | 1.000 | 0.909 | 0 | 9 | 0.000 | 10.000 | 1.000 |
| IL-1β | 1.000 | 0.978 | 1.000 | 2 | 10 | 0.002 | 9.800 | 0.980 |
| IL-2 | 1.000 | 0.978 | 1.000 | 2 | 10 | 0.002 | 9.800 | 0.980 |
| IL-22 | 1.000 | 0.978 | 1.000 | 2 | 10 | 0.002 | 9.800 | 0.980 |
| IL-4 | 1.000 | 0.978 | 1.000 | 2 | 10 | 0.002 | 9.800 | 0.980 |
| IL-5 | 1.000 | 0.978 | 1.000 | 2 | 10 | 0.002 | 9.800 | 0.980 |
| IL-6 | 1.000 | 0.000 | 1.000 | 0 | 1 | 0.000 | 1.000 | 0.000 |
| TNF-α | 1.000 | 0.978 | 1.000 | 2 | 10 | 0.002 | 9.800 | 0.980 |

IL, interleukin; IFN, interferon; TNF, tumor necrosis factor; CRP, C-reactive protein
