# Supplementary material for: Complex interaction networks of cytokines after transarterial chemotherapy in patients with hepatocellular carcinoma
Source: PLoS One. 2019 Nov 21;14(11):e0224318. doi: 10.1371/journal.pone.0224318 (PMC6874208; doi:10.1371/journal.pone.0224318)
Supplement: S12 Table — (DOCX) [file pone.0224318.s012.docx]

S12 Table. Topological parameters from network analysis of D60

|  | Average shortest | Clustering | Closeness | Stress | Degree | Betweenness | Neighborhood | Topological |
| --- | --- | --- | --- | --- | --- | --- | --- | --- |
|  | path length | coefficient | centrality |  |  | centrality | connectivity | coefficient |
| IFN-γ | 1.273 | 0.964 | 0.786 | 2 | 8 | 0.003 | 9.375 | 0.852 |
| IL-10 | 1.000 | 0.691 | 1.000 | 34 | 11 | 0.203 | 7.909 | 0.782 |
| IL-12 | 1.364 | 0.952 | 0.733 | 2 | 7 | 0.003 | 9.429 | 0.857 |
| IL-13 | 1.182 | 0.889 | 0.846 | 8 | 9 | 0.011 | 9.000 | 0.818 |
| IL-17α | 1.182 | 0.889 | 0.846 | 8 | 9 | 0.012 | 9.000 | 0.818 |
| IL-1β | 1.091 | 0.844 | 0.917 | 14 | 10 | 0.021 | 8.700 | 0.791 |
| IL-2 | 1.182 | 0.889 | 0.846 | 8 | 9 | 0.012 | 9.000 | 0.818 |
| IL-4 | 1.091 | 0.844 | 0.917 | 14 | 10 | 0.021 | 8.700 | 0.791 |
| IL-5 | 1.364 | 1.000 | 0.733 | 0 | 7 | 0.000 | 9.571 | 0.870 |
| IL-6 | 1.909 | 0.000 | 0.524 | 0 | 1 | 0.000 | 11.000 | 0.000 |
| IL-9 | 1.364 | 0.952 | 0.733 | 2 | 7 | 0.003 | 9.429 | 0.857 |
| TNF-α | 1.091 | 0.844 | 0.917 | 14 | 10 | 0.021 | 8.700 | 0.791 |

IL, interleukin; IFN, interferon; TNF, tumor necrosis factor; CRP, C-reactive protein
