# Supplementary material for: Complex interaction networks of cytokines after transarterial chemotherapy in patients with hepatocellular carcinoma
Source: PLoS One. 2019 Nov 21;14(11):e0224318. doi: 10.1371/journal.pone.0224318 (PMC6874208; doi:10.1371/journal.pone.0224318)
Supplement: S13 Table — (DOCX) [file pone.0224318.s013.docx]

S13 Table. Univariate and multivariate analysis by Cox regression analysis for D0 and D3

|  |  | Univariate |  |  | Multivariate |  |
| --- | --- | --- | --- | --- | --- | --- |
| D0 | P value | Hazard ratio | 95% CI | P value | Hazard ratio | 95% CI |
| IL-12 | NS |  |  |  |  |  |
| IFN-γ | NS |  |  |  |  |  |
| IL-17α | NS |  |  |  |  |  |
| IL-2 | <0.001 | 1.001 | 1.000 - 1.001 | 0.012 | 1.003 | 1.001- 1.005 |
| IL-10 | <0.001 | 1.001 | 1.000 - 1.002 | NS |  |  |
| IL-9 | NS |  |  |  |  |  |
| IL-22 | NS |  |  |  |  |  |
| IL-6 | 0.002 | 1.012 | 1.005 - 1.020 | NS |  |  |
| IL-13 | NS |  |  |  |  |  |
| IL-4 | NS |  |  |  |  |  |
| IL-5 | <0.001 | 1.000 | 1.000 - 1.000 | NS |  |  |
| IL-1β | 0.033 | 1.002 | 1.000 - 1.005 | NS |  |  |
| TNF-α | NS |  |  |  |  |  |
| CRP | <0.001 | 1.017 | 1.012 - 1.021 | <0.001 | 1.017 | 1.013 - 1.022 |

|  |  | Univariate |  |  | Multivariate |  |
| --- | --- | --- | --- | --- | --- | --- |
| D3 | P value | Hazard ratio | 95% CI | P value | Hazard ratio | 95% CI |
| IL-12 | NS |  |  |  |  |  |
| IFN-γ | NS |  |  |  |  |  |
| IL-17α | 0.043 | 1.003 | 1.000 - 1.006 | NS |  |  |
| IL-2 | NS |  |  |  |  |  |
| IL-10 | NS |  |  |  |  |  |
| IL-9 | NS |  |  |  |  |  |
| IL-22 | NS |  |  |  |  |  |
| IL-6 | <0.001 | 1.004 | 1.004 - 1.012 | 0.03 | 1.012 | 1.001 - 1.022 |
| IL-13 | 0.001 | 1.006 | 1.003 - 1.009 | NS |  |  |
| IL-4 | NS |  |  |  |  |  |
| IL-5 | NS |  |  |  |  |  |
| IL-1β | NS |  |  |  |  |  |
| TNF-α | NS |  |  |  |  |  |
| CRP | 0.001 | 1.015 | 1.006 - 1.024 | 0.033 | 1.011 | 1.001 - 1.022 |
