# Supplementary material for: Complex interaction networks of cytokines after transarterial chemotherapy in patients with hepatocellular carcinoma
Source: PLoS One. 2019 Nov 21;14(11):e0224318. doi: 10.1371/journal.pone.0224318 (PMC6874208; doi:10.1371/journal.pone.0224318)
Supplement: S14 Table — (DOCX) [file pone.0224318.s014.docx]

S14 Table. Univariate and multivariate analysis by Cox regression analysis for D7 and D60

|  |  | Univariate |  |  | Multivariate |  |
| --- | --- | --- | --- | --- | --- | --- |
| D7 | P value | Hazard ratio | 95% CI | P value | Hazard ratio | 95% CI |
| IL-12 | NS |  |  |  |  |  |
| IFN-γ | NS |  |  |  |  |  |
| IL-17α | NS |  |  |  |  |  |
| IL-2 | NS |  |  |  |  |  |
| IL-10 | NS |  |  |  |  |  |
| IL-9 | NS |  |  |  |  |  |
| IL-22 | NS |  |  |  |  |  |
| IL-6 | NS |  |  |  |  |  |
| IL-13 | NS |  |  |  |  |  |
| IL-4 | NS |  |  |  |  |  |
| IL-5 | NS |  |  |  |  |  |
| IL-1β | NS |  |  |  |  |  |
| TNF-α | NS |  |  |  |  |  |
| CRP | 0.016 | 1.009 | 1.006 - 1.017 |  |  |  |

|  |  | Univariate |  |  | Multivariate |  |
| --- | --- | --- | --- | --- | --- | --- |
| D60 | P value | Hazard ratio | 95% CI | P value | Hazard ratio | 95% CI |
| IL-12 | 0.12 | 1.002 | 1.000 - 1.004 | NS |  |  |
| IFN-γ | NS |  |  |  |  |  |
| IL-17α | 0.013 | 1.002 | 1.001 - 1.004 | NS |  |  |
| IL-2 | NS |  |  |  |  |  |
| IL-10 | NS |  |  |  |  |  |
| IL-9 | NS |  |  |  |  |  |
| IL-22 | 0.29 | 1 | 1.000 - 1.001 | NS |  |  |
| IL-6 | 0.001 | 1.001 | 1.000 - 1.001 | NS |  |  |
| IL-13 | NS |  |  |  |  |  |
| IL-4 | NS |  |  |  |  |  |
| IL-5 | NS |  |  |  |  |  |
| IL-1β | 0.005 | 1.003 | 1.000 - 1.005 | NS |  |  |
| TNF-α | 0.016 | 1.002 | 1.000 - 1.003 | NS |  |  |
| CRP | <0.001 | 1.023 | 1.012 - 1.033 | <0.001 | 1.022 | 1.010 - 1.033 |
